# Patients with dyspepsia have impaired mucosal integrity both in the duodenum and jejunum: in vivo assessment of small bowel mucosal integrity using baseline impedance

**DOI:** 10.1007/s00535-019-01614-5

**Published:** 2019-08-29

**Authors:** Kenichiro Nakagawa, Ken Hara, Asma Fikree, Shahab Siddiqi, Philip Woodland, Atsushi Masamune, Qasim Aziz, Daniel Sifrim, Etsuro Yazaki

**Affiliations:** 1 Wingate Institute of Neurogastroenterology, Barts and The London School of Medicine and Dentistry, Queen Mary University of London, 26 Ashfield Street, Whitechapel, London, E1 AJ UK; 2 Division of General Surgery, Broomfield Hospital, Court Rd, Broomfield, Chelmsford, CM1 7ET UK; 3 Division of Gastroenterology, Tohoku University Graduate School of Medicine, 1-1 Seiryo-machi, Aobaku, Sendai, 980-8574 Japan; 4 Division of Gastroenterology, Hyogo College of Medicine, 1-1 Mukogawacho, Nishinomiya, 663-8501 Hyogo Japan

**Keywords:** Functional dyspepsia, Small bowel motility, Small bowel mucosal integrity

## Abstract

**Background:**

Recent studies reported that impaired proximal duodenal mucosa, assessed by duodenal biopsy, could play an important role in the development of dyspeptic symptoms. The aims of this study were (a) to develop a method to measure “in vivo” duodenal and jejunal baseline impedance (BI) and (b) to assess small bowel mucosal integrity in patients with functional dyspepsia (FD) and healthy controls (HC).

**Methods:**

We recruited 16 patients with FD and 15 HC. All subjects underwent ambulatory duodeno-jejunal manometry combined with impedance (HRM/Z), BI were determined by measuring impedance immediately after the passage of nocturnal migrating motor complex (MMC) phase IIIs.

**Results:**

The number of MMC phase IIIs in FD was significantly lower than that in HC (2.6 ± 1.4 vs 4.8 ± 1.7, *p* < 0.001). The BI in patients was significantly lower than that in HC in D1(164.2 ± 59.8 Ω in FD and 243.1 ± 40.5 Ω in HC, *p* = 0.0061), D2 (191.2 ± 34.1 and 256.5 ± 91.4 Ω, *p* = 0.01), D3 (214.0 ± 76.9 and 278.1 ± 45.3 Ω, *p* = 0.009), D4 (270.8 ± 54.2 and 351.8 ± 50.2 Ω, *p* < 0.001), and J1 (312.2 ± 55.4 and 379.3 ± 38.3 Ω, *p* = 0.001).

**Conclusions:**

This is the first study reporting the duodenal and jejunal BI in vivo. The results have shown significantly lowered BI in the proximal small intestine in patients with FD compared to HC. Furthermore it suggests that measurements of small bowel BI could be used as a biomarker for diagnosis and follow up of patients with FD.

**Electronic supplementary material:**

The online version of this article (10.1007/s00535-019-01614-5) contains supplementary material, which is available to authorized users.

## Introduction

Functional dyspepsia (FD) is a disorder defined by Rome IV criteria as the presence of chronic bothersome early satiety, postprandial fullness, epigastric pain or burning without any organic, systemic or metabolic disease that is likely to explain the symptoms [1]. FD is a common gastroduodenal disorder, affecting up to 15–20% of the general population [2] and is associated with significant negative impact on the quality of life [3].

Traditionally, pathophysiological factors underlying FD focused on gastric functional and/or structural abnormalities, including gastric acid hyper-secretion, impaired gastric accommodation, delayed gastric emptying and hyper-sensitivity to gastric distention and helicobacter pylori infection [4–8].

More recently, it has been proposed that another pathophysiological factor in FD can be an alteration in the duodenal mucosa [9–13]. Talley et al. reported an increased number of duodenal eosinophils and mast cells in patients with FD compared to controls [9] and suggested a role of low-grade inflammation in FD. More recent studies have reported that proximal duodenal mucosal biopsies from patients with FD showed lower transepithelial electrical resistance and increased mucosal permeability compared to those from healthy controls [10]. The authors suggested that impaired duodenal mucosal barrier function could facilitate the passage of luminal antigens through the epithelium, which may induce low-grade inflammation and would contribute to bothersome dyspeptic symptoms. Whether these mucosal abnormalities are restricted to the duodenum or they further affect the proximal small intestine is unknown.

So far, duodenal mucosal integrity has been assessed through analysis of biopsies “in vitro”. In recent years, attempts have been made to assess mucosal integrity in the esophagus “in vivo”. Intraluminal esophageal impedance is a technique to detect gastro-esophageal reflux. Impedance measurements in the absence of reflux or swallowing (baseline impedance) reflects the integrity of the esophageal mucosa [14]. Low baseline impedance in the esophagus is widely accepted as a surrogate marker of abnormal mucosal integrity [15–17].

We hypothesized that measurements of intestinal mucosal baseline impedance could be used to assess small bowel mucosal integrity “in vivo”.

The aims of this study were (1) to develop a method to measure “in vivo” duodenal and jejunal baseline impedance and (2) to assess small bowel mucosal integrity in patients with FD and healthy controls.

## Methods

### Subjects

We recruited a total of 16 patients (14 females and 2 males; mean age 42.1 ± 12.1 years) meeting Rome IV criteria for FD [1] and 15 healthy controls (7 females and 8 males; mean age 36.6 ± 11.5 years) at the Upper Gastrointestinal Physiology Unit of the Royal London Hospital, UK.

Patients were recruited on the basis of dyspeptic symptoms (bothersome postprandial fullness and epigastric pain) by Rome IV diagnostic questionnaire for adults. The severity of dyspeptic symptoms was scored using dyspeptic symptom score (DSS) [18]. In all FD patients, organic, systemic, or metabolic disease, likely to explain the symptoms were excluded by clinical and biochemical examination, ultrasound of the upper abdomen and esophago-gastro-duodenoscopy. Subjects with a history of abdominal surgery (other than appendicectomy), coeliac disease, or inflammatory bowel disease were excluded. Subjects had no intake of non-steroidal anti-inflammatory drugs (NSAIDs), corticosteroids or other immunosuppressive drugs in the preceding 6 months.

All healthy asymptomatic controls had both negative *Helicobacter pylori* infection by ^13^C urea breath test (Diabact UBT, Kibion, Uppsala, Sweden) and negative lactulose hydrogen breath test (LHBT).

The study protocol was approved by the ethics committee of the London – Central Research Ethics Committee (ref: 17/LO/0701) and written informed consent was obtained from all the subjects.

### Ambulatory duodena-jejunal high-resolution manometry and impedance (HRM/Z)

**Fig. 1:**
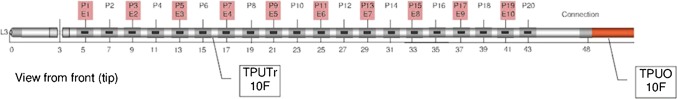
High-resolution manometry combined with impedance catheter. The high-resolution manometry combined with impedance catheter (UniSensor, Switzerland) comprises 20 pressure sensors spaced 2 cm apart and 9 pairs of impedance electrodes. *P* pressure sensor, *E* electrode, *TPUTr* thermoplastic polyurethane transparent, *TPUO* thermoplastic polyurethane orange

Duodeno-jejunal HRM/Z was recorded simultaneously using a dedicated ambulatory system (MMS, Version 9.2r, B.V.) and stored for subsequent display, and analysis. The HRM/Z catheter (UniSensor, Switzerland) comprises 20 pressure sensors spaced 2 cm apart and 9 pairs of impedance electrodes (Fig. 1).

**Fig. 2:**
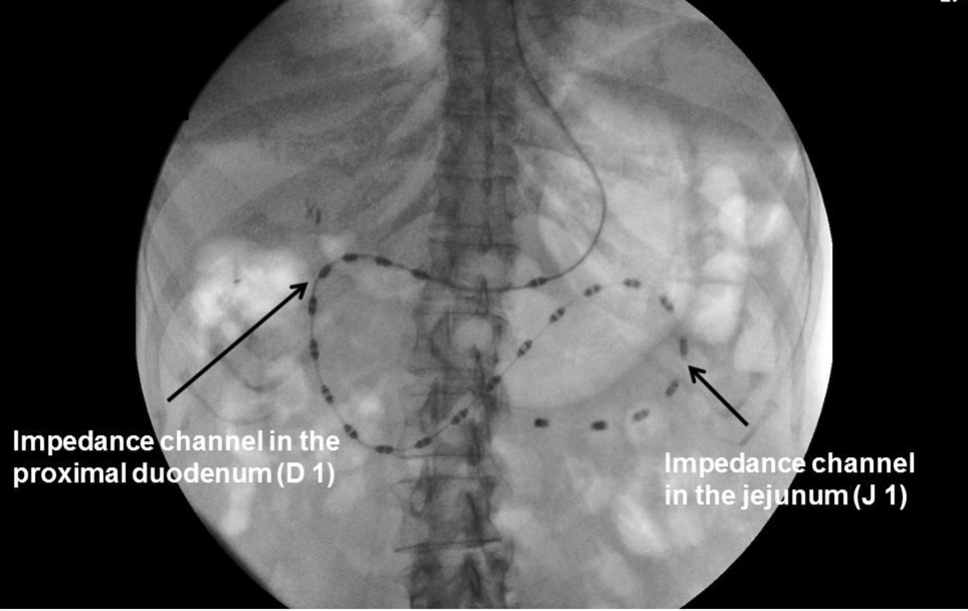
The position of catheter, pressure and impedance sensors. Pressure and impedance sensors were distributed from the antrum to the proximal jejunum

All subjects were asked to stop proton pump inhibitors for at least 1 week prior to the study. Subjects were fasted for at least 6 h before the intubation of the HRM/Z catheter. The catheter was inserted transnasally into the stomach, and its progression was monitored using fluoroscopic screening within the limited radiation dosage (0.2–0.4 mSv for each study) [19]. When the tip of the catheter was passed through the pylorus, a balloon attached to the tip of the catheter was inflated with 5 ml of air for further propulsion. The catheter was advanced until the tip was positioned beyond the ligament of Treitz and at least three pressure sensors remained in the gastric antrum. The balloon was then deflated. Figure 2 shows the position of the catheter. Pressure and impedance sensors were distributed from the antrum to the proximal jejunum.

After the intubation, HRM/Z recordings were started. Subjects were then given a standard meal (630 kcal, Fat 28 g, Carbs 77 g, Protein 19 g), and rested in a sitting position for 1 h. Recordings were continued in ambulatory settings. Subjects were allowed to have only water on day 1, and they were allowed to eat their typical breakfasts on the day 2. They returned to the hospital in the morning of the day 2, and the catheter was removed. A diary was provided to record their activities including timing of meals and sleeping.

### Detection of small intestinal bacterial overgrowth (SIBO) and *H. pylori* infection

LHBT was performed to assess SIBO. Subjects were asked to fast for 8–12 h and avoid fermentable foods such as complex carbohydrate 24 h prior to LHBT. Also, all subjects, if applicable, stopped antibiotics for at least 4 weeks and pro-motility drugs and laxatives at least one week prior to LHBT. After oral administration of 10 g of lactulose in 200 ml of water, breath samples were collected every 20 min for 120 min. A rise in hydrogen level of ≥ 20 ppm by 60 min was considered positive for SIBO [20].

*Helicobacter pylori* infection was assessed by ^13^C urea breath test. All subjects, if applicable, stopped acid suppressive medication for at least 2 weeks. After 8–12 h fasting period, breath samples were collected before and 10 min after the administration of ^13^C urea capsule with 200 ml water. *H. pylori* infection was considered to be negative if ^13^CO_2_ value was below a 2.5‰ level in the breath sample after 10 min [21, 22].

### Analysis of HRM/impedance recording

The manometric parameters were analyzed both semi-automatically (quantitative) and visually (qualitative). The pressure and impedance sensors in duodenum and jejunum could be fluoroscopically identified in D1, D2, D3, D4 and J1. The nocturnal and meal periods were identified based on diary entries. Automated analysis was initially performed for the identification of duodeno-jejunal contractile events [23]. A pressure event that exceeded a threshold of 10 mmHg, for which there was no simultaneous event occurring in the other channels, was assessed by the algorithm as being the consequence of an enteric contraction.

Phase III of the migrating motor complex (MMC) was defined as the presence of a period of phasic contractions that: (1) occurred for at least 2 min; (2) recurred at a frequency of 10–12 per min in duodenum and jejunum; (3) propagated ab-orally, as indicated by at least two recording sites and (4) was subsequently followed by a period of motor quiescence (phase I) [24–26].

The following parameters in proximal duodenum (D2) were calculated: (1) Duration of phase III; (2) Peak contraction amplitude of phase III; (3) MMC cycle period. The peak contraction amplitude of phase III was taken as the peak average amplitude of MMC in each subject. The MMC cycle period was taken as a period between the onset of phase III to the next onset of phase III.

### Baseline impedance measurement

In the small intestine, unlike in the esophagus, the mucosa is almost constantly covered by fluids, making it more difficult to assess the baseline mucosal impedance. We hypothesized that immediately after the passage of a phase III of the MMC, the intestinal segment is devoid of fluids and allows measurement of intestinal mucosal baseline impedance. The baseline impedance was obtained during nocturnal periods where artefacts were minimal.

**Fig. 3:**
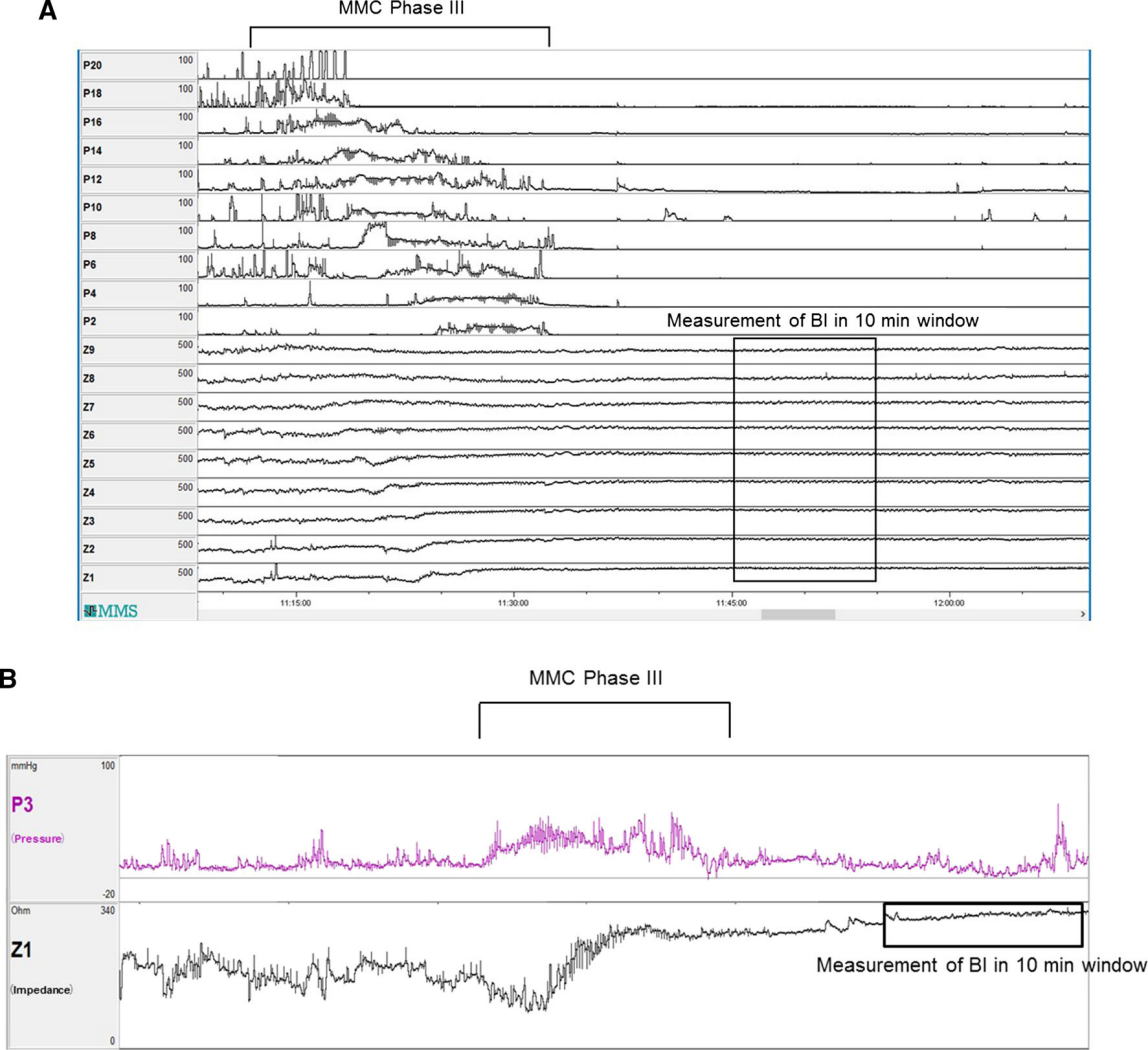
**a** Manometry and impedance traces at the timing of MMC pIII. *MMC pIII* migrating motor complex phase III, *BI* baseline impedance. **b** Example of measurement of baseline impedance. The mean baseline impedance was measured by taking an average impedance value of 10-min time windows after the passage of MMC phase III, where a plateau in impedance was visually identified. *MMC* migrating motor complex, *P3* pressure channel 3, *Z1* impedance channel 1, *BI* baseline impedance

The mean baseline impedance was measured by taking an average impedance value of 10-minute time windows after the passage of MMC phase III, where a plateau in impedance was visually identified (Fig. 3a, b).

### Statistical analysis

All data were expressed as mean ± standard deviation (SD). Single comparisons were made with an unpaired student’s *t* test (parametric data) or Mann–Whitney *U* test (nonparametric data) wherever appropriate. Correlations were tested using the Spearman and Pearson tests wherever appropriate. Fisher’s exact test was used to test proportional differences. Significance was declared at *p* < 0.05. Statistical analysis was performed with Microsoft Excel 2016 or JMP Pro 14 (SAS Institute, Cary, NC, USA).

## Results

All 16 patients with FD (14 females and 2 males; mean age 42.8 ± 11.8 years) and 15 healthy controls (HC) (7 females and 8 males; mean age 36.7 ± 11.5 years) completed the study. Seven patients with FD were diagnosed by Rome IV criteria as postprandial distress syndrome (PDS) and 3 were epigastric pain syndrome (EPS), 6 were overlapping PDS and EPS characteristics. Clinical characteristics of the patients were described in Table 1. There was no significant difference in age between patients and HC. The proportion of female in patients with FD was significantly higher than that in HC. Body mass index (BMI) in both groups was within the normal range. The number of *H. pylori* positive was 1/16 patient. Eight out of 16 patients underwent LHBT during the study periods. 1 out of 8 was positive for SIBO. Seven patients concomitant irritable bowel syndrome (IBS) symptoms. None of participants were on NSAIDs, corticosteroids or other immunosuppressive medications.

**Table 1:** Clinical characteristics

	FD *n* = 16	HC *n* = 15	*p* value
Age	42.8 (11.8)	36.7 (11.5)	N.S
Male/female	2/14	8/7	0.023
BMI	24.8 (3.2)	23.9 (2.9)	N.S
Dyspeptic symptom score	13.5 (4.4)	0 (0)	< 0.001
*H.pylori* positive/negative	1/15	0/15	–
LHBT positive/negative	1/7	0/15	–

Data is shown as mean ± SD

*FD* functional dyspepsia, *HC* healthy controls, *BMI* body mass index, *H. pylori* Helicobacter pylori, *LHBT* lactulose hydrogen breath test

### Manometric parameters

The total duration of the nocturnal periods in patients with dyspepsia and control was 8.19 ± 1.6 and 8.63 ± 1.1 h (N.S.), respectively. Table 2 summarizes the parameters characterizing nocturnal duodeno-jejunal MMC phase III contractions. All subjects had at least one complete MMC cycle recorded during nocturnal period. In total, 108 nocturnal MMC phase IIIs (mean 3.92 per subject, SD 1.96) were identified. The number of MMC phase IIIs in patients was significantly lower than that in HC (2.6 ± 1.4 vs 4.8 ± 1.7, *p* < 0.001). The average interval of MMC cycle in FD was significantly longer than that in HC (153.4 ± 85.8 vs 81.1 ± 21.4 min, *p* = 0.004). There were no statistical differences in the duration of MMC phase III and the peak amplitude between the two groups (5.6 ± 2.6 vs 5.1 ± 1.7 min, N.S; 82.3 ± 16.8 vs 82.0 ± 24.7 mmHg, N.S, respectively).

**Table 2:** The manometric parameters (D2)

	FD *n* = 16	HC *n* = 15	*p* value
The number of MMC pIII	41	67	–
The number of MMC pIII/patient	2.6 (1.4)	4.8 (1.7)	< 0.001
The duration of MMC pIII (min)	3.8 (1.4)	4.4 (1.3)	N.S
The average of peak amplitude (mmHg)	82.3 (16.8)	82.0 (24.7)	N.S
The average duration of MMC cycle (min)	148.4 (82.1)	85.8 (18.4)	0.008

Data is shown as mean ± SD

*FD* functional dyspepsia, *HC* healthy controls, *MMC pIII* migrating motor complex phase III

### Duodeno-jejunal baseline impedance

**Fig. 4:**
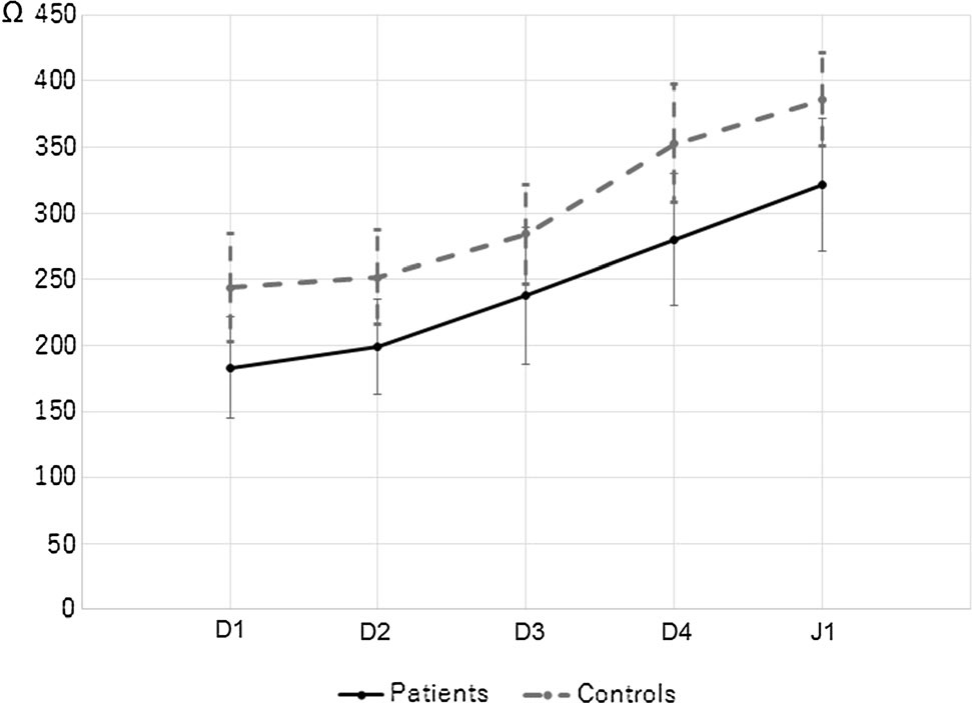
Differences in duodeno-jejunal baseline impedance in FD and HC. *FD* functional dyspepsia, *HC* healthy controls

Duodeno-jejunal baseline impedance values in each segment (D1, D2, D3, D4, J1) in patients and HC were shown in Table 3 and graphically in Fig. 4. The baseline impedance increased from D1 to J1 in both FD and HC group. The baseline impedance in patients was significantly lower than that in HC in D1 (164.2 ± 59.8 Ω in FD and 243.1 ± 40.5 Ω in HC, *p* = 0.0061), D2 (191.2 ± 34.1 and 256.5 ± 91.4 Ω, *p* = 0.01), D3 (214.0 ± 76.9 and 278.1 ± 45.3 Ω, *p* = 0.009), D4 (270.8 ± 54.2 and 351.8 ± 50.2 Ω, *p* < 0.001), and J1 (312.2 ± 55.4 and 379.3 ± 38.3 Ω, *p* = 0.001). Also, there was no statistical difference in baseline impedance between female and male in D1 (254.1 ± 43.6 and 228.4 ± 38.9 Ω, N.S.), in D2 (275.6 ± 128.8 and 239.8 ± 42.6 Ω, N.S.), in D3 (277.7 ± 47.1 and 278.4 ± 46.8 Ω, N.S.), in D4 (356.8 ± 48.5 and 347.4 ± 54.6 Ω, N.S.), and J1 (369.1 ± 47.7 and 388.3 ± 28.1 Ω, N.S.)

**Table 3:** Baseline impedance after MMC pIII

Segment	FD *n* = 16	HC *n* = 15	*p* value
D1	164.2 (59.8)	243.1 (40.5)	0.006
D2	191.2 (34.1)	256.5 (91.4)	0.01
D3	214.0 (76.9)	278.1 (45.3)	0.009
D4	270.8 (54.2)	351.8 (50.2)	< 0.001
J1	312.2 (55.4)	379.3 (38.3)	0.001

Data is shown as mean ± SD

*MMC pIII* migrating motor complex phase III, *FD* functional dyspepsia, *HC* healthy controls

### The correlation between baseline impedance and the number of MMC phase III contractions

There were weak positive correlations between baseline impedance and the number of MMC phase III contractions in J1 (*R* = 0.22, *p* = 0.014). However, no correlations were shown in D1, D2, D3 and D4.

### The correlation between baseline impedance and severity of symptoms

Severity of dyspeptic symptoms were assessed using DSS.

There were no statistical correlations between baseline impedance in the each segment and severity of symptoms (D1, *R* = 0.07, N.S; D2, *R* = 0.01, N.S; D3, *R* = 0.01, N.S; D4, *R* = 0.04, N.S; J1, *R* = 0.03, N.S.)

## Discussion

Dyspeptic symptoms significantly impact on daily life. The causes of these symptoms, such as postprandial fullness, early satiety, epigastric discomfort/pain and burning, are not fully explained [1, 27]. However, recent studies reported that low-grade inflammation in the proximal duodenum and impaired proximal duodenal mucosal integrity, assessed by duodenal biopsy, could play an important role in the development of dyspeptic symptoms [9, 10]. Cirillo et al. reported neuronal functional abnormalities and altered ganglionic architecture in the duodenal submucous plexus in biopsies from patients with FD [28]. They suggested that low grade inflammation induced by impairment of intestinal barrier function may affect specific neuronal pathways underlying dyspeptic symptoms such as early satiety and postprandial fullness. Miwa et al. also proposed the possibility that the duodenum of patients with FD is more sensitive to noxious stimuli because of low-grade inflammation and increased mucosal permeability, and gastric motility abnormalities and gastric hypersensitive might be induced by stimulation of the duodenum [12]. In this study, we have assessed “in vivo” the integrity of duodenal and jejunal mucosa using, for the first time, measurements of baseline impedance during ambulatory duodeno-jejunal HRM-impedance monitoring.

To measure duodeno-jejunal baseline impedance, we had to simultaneously measure small intestinal motility and impedance, and identify phase III of the migrating motor complex. By doing so, we have also found that patients with FD have decreased number of phase III contractions of the MMC.

Small bowel manometry has been regarded as one of the clinical investigation tools to evaluate functional gastro-intestinal (GI) disorders. Vantrappen et al*.* have reported that the MMC phase III regulated by enteric nerve system is important in helping to maintain fasting aboral transit and low bacterial counts in the small intestine [26]. MMC phase III is therefore thought to be a housekeeping phenomenon clearing the gastrointestinal contents in digestive processes. In the present study, manometric finding showed the number of nocturnal MMC phase IIIs in patients with FD was significantly lower than that in HC. This result was in agreement with previous reports by Jebbink et al. [29] and Wilmer et al. [30]. They demonstrated, using ambulatory manometry technique, that MMC cycles in patients with FD occurred less frequently than in control group and suggested that this reduced incidence of MMC cycle could lead to delayed interdigestive transit then might cause dyspeptic symptoms. Also, Jacobs et al. suggested that impaired MMC phase III can cause SIBO [31]. LHBT was performed only in 8 out of 16 patients with FD. It may be therefore difficult to discuss the possible correlation between SIBO and MMC phase III. We showed that there was a weak but positive correlation between the nocturnal number of MMC phase III and baseline impedance in the proximal jejunum. This might suggest that reduced phase III leads to prolonged exposure of the jejunum to luminal contents and hence mucosal damage could occur. Further study will be needed to assess the relationship between jejunal impedance and intestinal motility.

The usage of ambulatory manometry together with impedance recordings provide the information of not only motor activity but possibly mucosal status as expressed by the baseline impedance value. To our knowledge this study has shown, for the first time, significantly lower baseline impedance from the duodenum to the proximal jejunum in patients with FD when compared to HC. The relationship between low basal impedance and symptoms is not completely clear. In the esophagus, patients with lower baseline impedance have higher esophageal sensitivity to acid exposure [32]. It is possible that similar relationship occurs in the intestine. We did not show a correlation between severity of symptoms and baseline impedance values. We should acknowledge however that perception of dyspeptic symptoms is likely to be a consequence of a complex pathophysiological cascade from intestine to central nervous system, and symptom questionnaires usually used to assess patients with FD are unlikely to be sensitive enough to detect the isolated role of impaired mucosal integrity.

Like esophageal mucosal integrity in non-erosive reflux disease, a low baseline impedance in the proximal small intestine (in the absence of endoscopic findings) could be used as a biomarker to identify patients with proximal functional GI disorders and theoretically to evaluate the outcome of treatment. However, further studies are needed to clarify whether the baseline impedance can indeed recover after treatments with acid suppression therapy [33, 34], prokinetic drugs [35] and/or acotiamide [36, 37].

In this study, a gradual increase in baseline impedance from D1 to J1 was observed in both patients with FD and HC. These impedance changes could be explained in two ways. Firstly, structural/anatomical differences of intestinal villus and tight junctions from the proximal duodenum to jejunum may affect the baseline impedance values. Secondly, duodenal mucosa could have more direct burden due to several digestive enzymes such as pepsin, hydrochloric acid as gastric juice and trypsin, amylase and lipase as pancreatic juice, which may affect the proximal duodenum most, and those chemical impacts could gradually be fading towards the jejunum.

In our patients, we found impaired mucosal integrity not only in the duodenum (as previously reported using biopsies), but also in the jejunum. FD and IBS are the two most prevalent functional gastrointestinal disorders and they might have overlapping pathophysiological mechanisms such as increased mast cell and intraepithelial lymphocyte concentrations, and increased paracellular intestinal permeability [38, 39]. It is possible, therefore that our finding of jejunal mucosal impairment in patients with FD could be due to concomitant IBS. However, our FD patients without IBS symptoms (*n* = 9), still had low jejunal baseline impedance compared to controls (see supplementary Table 1 and supplementary Figure 1).

The following limitations of our study are acknowledged. We did not perform microscopic assessment of mucosal changes to investigate mucosal barrier function. Our study therefore does not provide a correlation between duodenal baseline impedance and in vitro measurements of duodenal mucosa in using chambers. However, previous studies have already described that impaired duodenal mucosal integrity and permeability using biopsy sample [10] in patients with FD, and increased mucosal admittance through endoscopic technique [40] in FD compared to HC. This study did not show a significant statistical correlation between baseline impedance and severity of dyspeptic symptoms. A study using increased numbers of FD patients with wider symptom severity would further assess this possible correlation.

In conclusion, this is the first study reporting the duodenal and jejunal baseline impedance in vivo. The results have shown significantly lowered baseline impedance in the proximal small intestine in patients with FD compared to HC. These findings confirm previous “in vitro” assessments. This suggests that impaired small bowel mucosal integrity may play an important role in pathophysiology of FD. Furthermore it suggests that, as techniques are refined, measurements of small bowel baseline impedance could theoretically be used as a biomarker for diagnosis and follow up of patients with FD.

## Electronic supplementary material

Below is the link to the electronic supplementary material.

Supplementary file1 (DOCX 12 kb)

Supplementary file2 (DOCX 13 kb)

Supplementary file3 (JPG 45 kb)

## Abbreviations

DSS: Dyspeptic symptom score
EPS: Epigastric pain syndrome
FD: Functional dyspepsia
HC: Healthy controls
HRM/Z: High-resolution manometry and impedance
IBS: Irritable bowel syndrome
LHBT: Lactulose hydrogen breath test
MMC: Migrating motor complex
NSAIDs: Non-steroidal anti-inflammatory drugs
PDS: Postprandial distress syndrome
SIBO: Small bowel bacterial overgrowth
GI: Gastro-intestinal
