# Supplementary material for: Patients with dyspepsia have impaired mucosal integrity both in the duodenum and jejunum: in vivo assessment of small bowel mucosal integrity using baseline impedance
Source: J Gastroenterol. 2019 Aug 29;55(3):273–80. doi: 10.1007/s00535-019-01614-5 (PMC7026227; doi:10.1007/s00535-019-01614-5)
Supplement: Supplementary file 1 — Supplementary file1 (DOCX 12 kb) [file 535_2019_1614_MOESM1_ESM.docx]

**Supplementary figure 1 Differences in duodeno-jejunal baseline impedance in FD and HC (IBS negative)**

The baseline impedance in FD patients without IBS symptoms was significantly lower than that in HC in D1(156.0 ± 84.1 Ω in FD and 243.1 ± 40.5 Ω in HC, p=0.033), D2 (173.2 ± 29.0 and 256.5 ± 91.4 Ω, p=0.011), D3 (177.6 ± 83.7 and 278.1 ± 45.3 Ω, p<0.001), D4 (246.8 ± 51.0 and 351.8 ± 50.2 Ω, p<0.001), and J1 (289.5 ± 65.4 and 379.3 ± 38.3 Ω, p=0.006).

FD, functional dyspepsia; HC, healthy controls; IBS, irritable bowel syndrome;
