# Supplementary material for: Patients with dyspepsia have impaired mucosal integrity both in the duodenum and jejunum: in vivo assessment of small bowel mucosal integrity using baseline impedance
Source: J Gastroenterol. 2019 Aug 29;55(3):273–80. doi: 10.1007/s00535-019-01614-5 (PMC7026227; doi:10.1007/s00535-019-01614-5)
Supplement: Supplementary file 2 — Supplementary file2 (DOCX 13 kb) [file 535_2019_1614_MOESM2_ESM.docx]

【Supplementary table 1】Baseline impedance after MMC pIII (IBS negative)

| Segment | FD n=9 | HC n=15 | P value |
| --- | --- | --- | --- |
| D1 | 156.0 (84.1) | 243.1 (40.5) | 0.033 |
| D2 | 173.2 (29.0) | 256.5 (91.4) | 0.011 |
| D3 | 177.6 (83.7) | 278.1 (45.3) | <0.001 |
| D4 | 246.8 (51.0) | 351.8 (50.2) | <0.001 |
| J1 | 289.5 (65.4) | 379.3 (38.3) | 0.006 |

Data is shown as mean ± SD.

MMC pIII, migrating motor complex phase III; IBS, irritable bowel syndrome; FD, functional dyspepsia; HC, healthy controls;
