# Supplementary figures and images for: Patients with dyspepsia have impaired mucosal integrity both in the duodenum and jejunum: in vivo assessment of small bowel mucosal integrity using baseline impedance
Source: J Gastroenterol. 2019 Aug 29;55(3):273–80. doi: 10.1007/s00535-019-01614-5 (PMC7026227; doi:10.1007/s00535-019-01614-5)

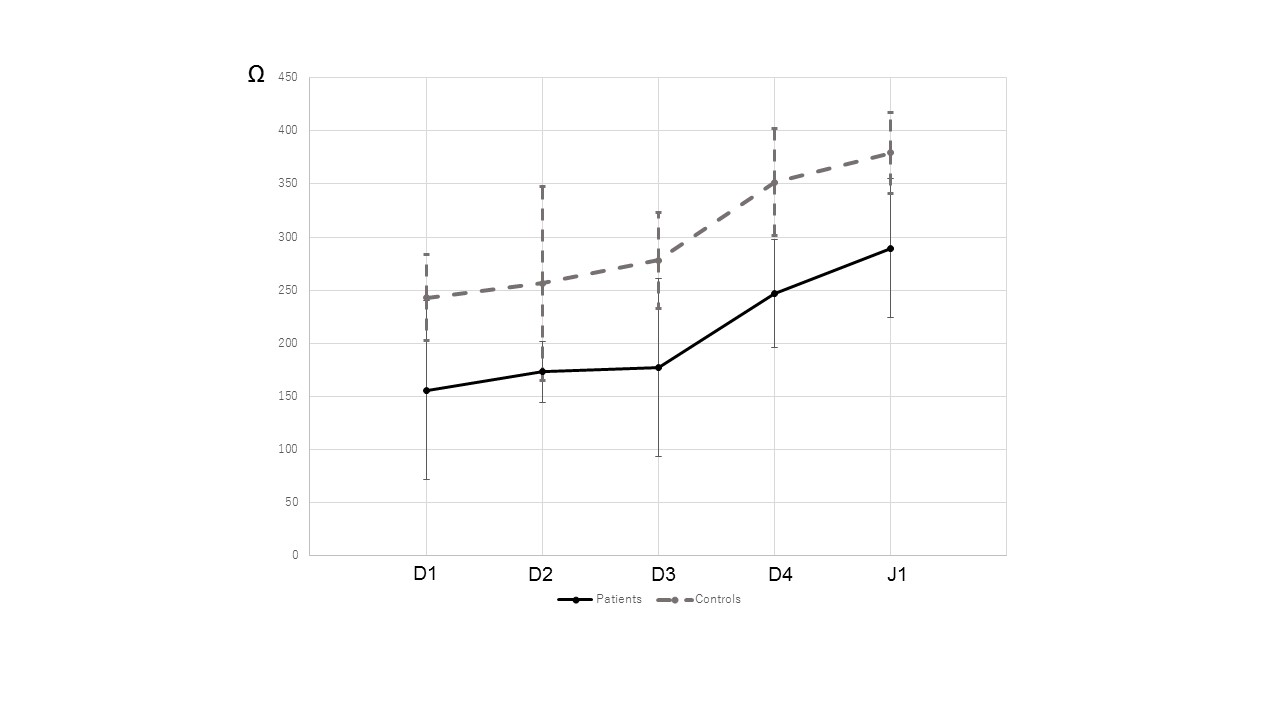

Supplement: Supplementary file 3 — Supplementary file3 (JPG 45 kb) [file 535_2019_1614_MOESM3_ESM.jpg]
